# All-cause excess mortality observed by age group and regions in the first wave of the COVID-19 pandemic in England

**DOI:** 10.2807/1560-7917.ES.2020.25.28.2001239

**Published:** 2020-07-16

**Authors:** Mary A Sinnathamby, Heather Whitaker, Laura Coughlan, Jamie Lopez Bernal, Mary Ramsay, Nick Andrews

**Affiliations:** 1 Public Health England, London, United Kingdom

**Keywords:** all-cause mortality, EuroMOMO, COVID-19 pandemic

## Abstract

England has experienced one of the highest excess in all-cause mortality in Europe during the current COVID-19 pandemic. As COVID-19 emerged, the excess in all-cause mortality rapidly increased, starting in March 2020. The excess observed during the pandemic was higher than excesses noted in the past 5 years. It concerned all regions and all age groups, except the 0–14 year olds, but was more pronounced in the London region and in those aged ≥ 85 years.

Excess all-cause mortality has traditionally been noted during the winter months in England, with extreme cold weather and circulation of seasonal influenza, being some of the main contributors for winter excesses [1-4]. High excess mortality levels were previously observed in England in the 2014/15 and 2017/18 seasons, which coincided with influenza seasons dominated by influenza A(H3N2), a subtype known to affect the older population (≥ 65 years of age) [2,4].

The 2019/20 influenza season has been dominated by the circulation of influenza A(H3N2), with an early start (breaching expected levels in week 47, 18–24 November, 2019) [5]. Excess mortality was observed when influenza activity was at its highest but subsequently decreased below expected levels in mid-January [6]. Subsequently, in late January, the first cases of coronavirus disease (COVID-19) were detected. A rapid increase in excess all-cause mortality was observed approximately 2 weeks after the announcement of the first COVID-19 confirmed death on 5 March 2020.

This study describes the exceptionally high all-cause excess mortality which has been observed during the COVID-19 pandemic in England, up to calendar week 20 (week ending 17 May 2020).

## Monitoring excess all-cause mortality

All-cause excess mortality is routinely monitored in England, using the European monitoring of excess mortality (EuroMOMO) algorithm, which was initiated to allow for timely comparisons across European countries for public health action [7]. The algorithm uses observed weekly deaths, by week of death from the past 5 years, and applies a correction for delay from death to registration based on historic delays. A baseline expected count is calculated from a Serfling model with a trend fitted using an over-dispersed Poisson distribution and the excess and corresponding standard deviations (z-scores) above the baseline calculated [8].

Daily aggregated all-cause death registrations in England are collected from the General Registry Office including information on date of death, date of registration, sex, age and Public Health England (PHE) regions. The analysis in this report is based on registrations until 02 June 2020 and shows deaths until the end of week 20 2020 (17 May 2020).

The following age groups were used: 0–14 years, 15–44 years, 45–64 years, 65–74 years, 75–84 years and ≥ 85 years. The data were also examined by the four PHE regions [9].

Cumulative excess all-cause mortality rates were calculated using annual population estimates from the Office for National Statistics (ONS) [10].

All statistical analyses were carried out in STATA v15.1 (StataCorp, TX).

## Estimates of excess all-cause mortality

**Figure 1:**
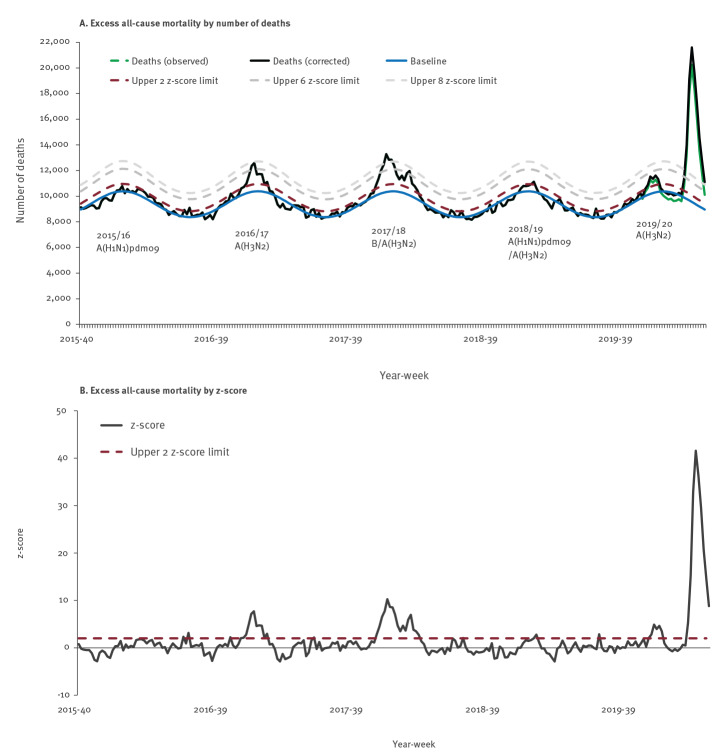
Overall excess all-cause mortality in all ages (A) by number of deaths and predominant circulating influenza subtype and (B) by z-score, calendar week 40 2015 to 20 2020

England has experienced much higher all-cause excess mortality in the 2019/20 season when comparing excess mortality observed in the past 5 years (Figure 1).

All age all-cause mortality increased rapidly, exceeding expected levels from week 12 2020 and peaking in week 15 2020 (with 12,045 estimated excess deaths and a z-score of 41.6). An amount of 56,456 all-cause excess deaths was estimated between week 12 2020 and week 20 2020 (Figure 1). The cumulative all-cause excess mortality rate between weeks 10 and 20 was 100.83 per 100,000 population with an observed-to-expected mortality ratio of 1.54, exceeding cumulative rates and ratios observed in any full seasons (Table).

**Table:** Cumulative estimated all-cause excess mortality rates per 100,000 population and observed/expected mortality ratios, calendar week 40 2015–week 20 2020

Criteria	Crude excess mortality rate (observed/expected ratio) in weeks 40 to 20 each season					
	2015/16	2016/17	2017/18	2018/19	2019/20: week 40 to 09	2019/20: week 10 to 20
Age group						
All ages	0.95 (1.00)	21.72 (1.04)	48.73 (1.08)	−2.65 (1.00)	13.20 (1.02)	100.83 (1.54)
0–14 years	0.25 (1.01)	0.90 (1.04)	0.45 (1.02)	0.31 (1.02)	0.74 (1.06)	0.33 (1.05)
15–44 years	−1.78 (0.96)	−0.24 (0.99)	1.82 (1.04)	0.41 (1.01)	−1.34 (0.95)	5.79 (1.46)
45–64 years	2.82 (1.01)	0.62 (1.00)	15.25 (1.06)	2.52 (1.01)	3.89 (1.02)	41.08 (1.47)
65–74 years	0.83 (1.00)	25.05 (1.03)	58.87 (1.06)	6.34 (1.01)	8.69 (1.01)	131.65 (1.42)
75–84 years	32.06 (1.01)	98.98 (1.03)	205.01 (1.07)	−17.16 (0.99)	77.19 (1.04)	508.77 (1.58)
≥ 85 years	−18.87 (1.00)	554.24 (1.06)	1,061.71 (1.11)	−160.23 (0.98)	280.17 (1.04)	1,767.16 (1.59)
PHE regions						
North	3.24 (1.00)	21.75 (1.03)	48.80 (1.08)	−9.09 (0.99)	11.68 (1.03)	105.49 (1.50)
Midlands and East	−1.91 (1.00)	22.07 (1.04)	48.51 (1.09)	−1.24 (1.00)	15.39 (1.04)	92.00 (1.53)
London	3.19 (1.01)	15.70 (1.04)	33.72 (1.09)	4.69 (1.01)	12.80 (1.05)	122.77 (2.06)
South	5.24 (1.01)	24.16 (1.04)	51.19 (1.08)	−5.76 (0.99)	16.79 (1.04)	94.89 (1.44)

PHE: Public Health England.

**Figure 2:**
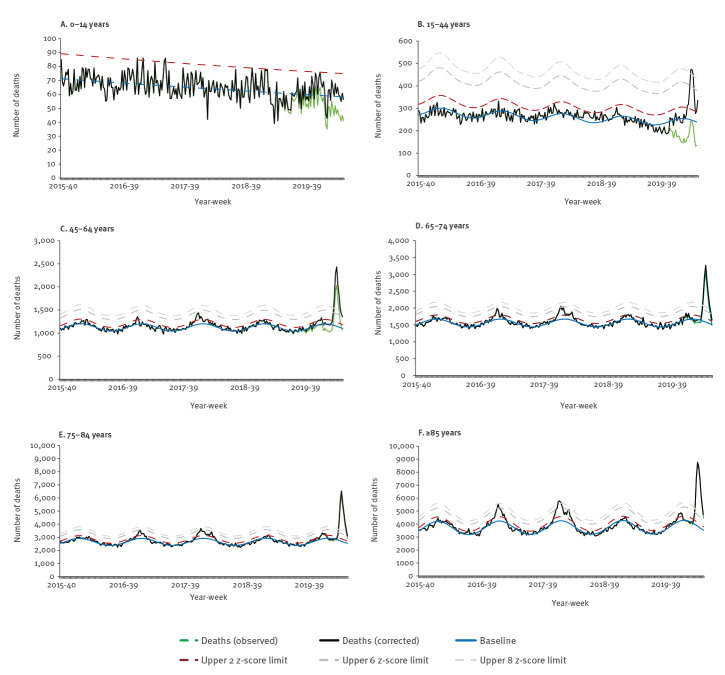
Excess all-cause deaths by age groups, England, calendar week 40 2015–week 20 2020

By age group, high levels of excess mortality, exceeding 8 z-scores above baselines, were evident in all but the 0–14 year olds. The highest excess in deaths was observed in those ≥ 85 years old, with cumulative mortality rates in the latter weeks of the 2019/20 season, exceeding those observed in the past 5 years including the 2017/18 season (Figure 2). Decreases in deaths since the peak week have been slow in this age group compared with decreases in other age groups. Similar observations were seen in the 75–84 year olds but with more rapid decreases from the peak. Observed and delay-corrected numbers of deaths were similar in older age (≥ 65 years of age) groups, but in younger age groups a substantial correction is required for many months due to the fact that many deaths are referred to a Coroner.

**Figure 3:**
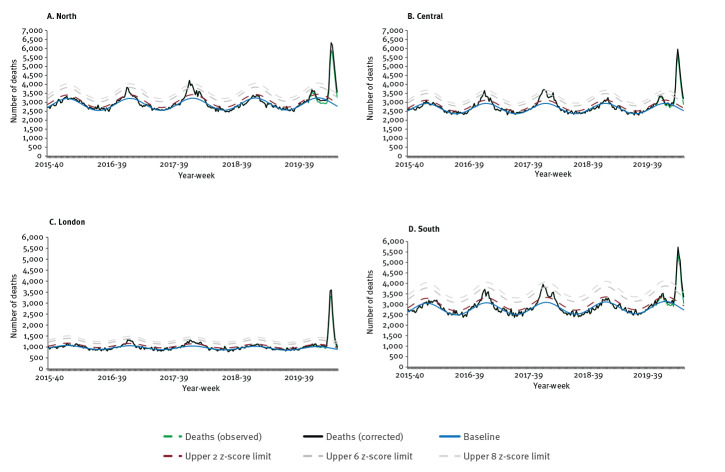
Excess all-cause deaths by regions, England, calendar week 40 2015–week 20 2020

All regions exceeded expected levels from week 12 2020, with a peak in week 15 2020. London observed the highest excess mortality rate (122.77/100,000) since the 2015/16 season and subsequently returned close to expected levels faster than the other regions by week 20, with other regions remaining above the upper 6 z-score limit at week 20 (Figure 3 and Table).

## Discussion

This study describes the very high all-cause excess mortality observed in England during the current COVID-19 pandemic. The findings show that England has experienced the highest excess all-cause mortality in comparison to previous seasons since 2015/16. The excess appears to be higher than that reported in other European countries based on z-score analyses [11].

At the beginning of the winter of 2019/20, England observed low excess all-cause mortality in comparisons to previous seasons which is unusual for a winter dominated by the circulation of influenza A(H3N2). The high number of all-cause deaths observed in week 15 across all ages except the 0–14 year olds, occurred 2 weeks after the ‘Stay at home’ lockdown government guidelines were enforced on 23 March 2020 (week 13) [12]. This is also in line with the peak in deaths due to COVID-19 in England reported elsewhere [13,14].

We found that excess mortality was higher in the oldest population, ≥ 85 year olds, with cumulative mortality rates being the highest observed in the past 5 years. This coincides with this age group also experiencing the highest case detections and hospitalisations as well as higher COVID-19-confirmed deaths as a proportion of overall cases [14]. Studies in other European countries have found the same higher rates in this and other older age groups [15,16].

A recent study investigating the relative risk of COVID-19 deaths in the United States and Europe suggests that the < 65 years age group has the lowest risk [17]. This is in line with our findings of no or low excess mortality observed in the 0–14 year olds, also noted in another recent European report [18].

We present all-cause excess mortality by regions in England. London was observed to have the highest excess all-cause mortality in comparison to other regions and its own historical trends but was also the region where rapid decreases were noted despite having the highest number of cases by region [14]. This also coincides with London being the region with the highest number of cases and seroprevalence [14]. Similar regional differences have been reported by other European countries and linked to regionally higher numbers of COVID-19 deaths [16].

One of the main strengths of this study is the use of the standardised EuroMOMO algorithm, which allowed us to make comparisons with other European countries and across seasons for many years. The need for such rapid assessment of excess mortality has been advocated during this pandemic [19]. A limitation of the z-score analysis however is that it does not allow a clear comparison of the exact scale of the excess between countries, because z-scores do not translate to the same number or proportional excess in different countries.

Further work will be carried out to examine the contribution of cause-specific deaths to the overall excess and understanding the contributing factors to the high excess mortality seen in all adults in England.

Overall, our findings highlight the importance of monitoring excess all-cause mortality as an indicator of disease burden in situations such as the current pandemic caused by a previously-unknown pathogen, for which the impact is still difficult to assess at the present time. Continued excess mortality monitoring will help assess the effect of relaxation of lockdown measures and mortality excess that may not be directly related to COVID-19 infections.
